# Bimodal Cell Size and Fusing Cells Observed in a Clonal Culture of the Ciguatoxin-Producing Benthic Dinoflagellate *Gambierdiscus* (WC1/1)

**DOI:** 10.3390/toxins14110767

**Published:** 2022-11-07

**Authors:** Michael J. Holmes, Richard J. Lewis

**Affiliations:** Institute for Molecular Bioscience, The University of Queensland, Brisbane 4072, Australia

**Keywords:** *Gambierdiscus*, ciguatera, cell fusion, possible sexual reproduction, mixotrophy, ciguatoxin, maitotoxin-3

## Abstract

Cells in a clonal culture of the WC1/1 strain of *Gambierdiscus* that produced ciguatoxin and maitotoxin-3 were observed to spontaneously fuse during the light phase of culture growth. Cells in the process of fusion were indistinguishable from other cells under the light microscope, except that at least one (often both) of the fusing cells displayed an extendible, finger-like protrusion (presumed peduncle) arising from near the sulcul region. Fusion started with one of the cells turning 90° to place the planes of the girdles approximately at right angles to each other, and movement of the transverse flagella ceased in both cells, or in the cell seen in girdle (lateral) view. The cell in girdle view appeared to fuse into the theca of the other cell. The cell that had turned 90° often rounded up and become egg shaped (obovoid) during early fusion. Fusion can be quick (<10 min) or can take more than an hour. We saw no evidence of the theca being shed during fusion. Measurement of the dorsoventral and transdiameters revealed a wide range for cell sizes that were distributed as a bimodal population in the clonal culture. This bimodal cell population structure was maintained in clonal cultures reisolated from a small or large cell from the original WC1/1 culture. Cellular production of ciguatoxins by the WC1/1 clone increased during the first two years in culture with a corresponding decrease in production of maitotoxin-3, but this inverse relationship was not maintained over the following ~1.5 years.

## 1. Introduction

*Gambierdiscus* is a genus of marine, benthic dinoflagellates that produce a wide array of polyether toxins, including ciguatoxins that accumulate through marine food chains to cause the disease ciguatera [1,2,3]. *Gambierdiscus* also produces a variety of highly toxic maitotoxins as well as lower toxicity polyether compounds, including gambieric acids, gambierone, and 44-methylgambierone [4,5,6,7,8,9,10,11,12,13,14]. The genus was initially described as a single species, *G. toxicus*, isolated from the Gambier Islands in French Polynesia [15] but 18 species have since been recognized [1]. Cultures are necessary to study many aspects of the biology of microalgae, including benthic dinoflagellates; however, cell morphology can change in culture from that seen in the wild. For example, cultured *Gambierdiscus* can sometimes grow with a “warty” abnormal morphology that can make them unrecognizable [5,16,17], with the abnormal morphology sometimes induced by growth in high nutrient concentrations [5]. The warty appearance of dinoflagellates has been long attributed to morphological aberrations of vegetative cells or zygotes produced by sexual reproduction [18].

Sexual reproduction by *Gambierdiscus* was first suggested by Taylor [19], after observing early fusion stages. More recently, Bravo et al. [20] described gamete pairs of *Gambierdiscus* from a clonal culture producing a large range of cell sizes, although they did not observe gamete fusion. Subsequent studies suggested that non-optimal temperatures promote sexuality in this genus, however, the processes in the sexual lifecycle of *Gambierdiscus* remain unknown [21].

An Australian clone of *Gambierdiscus* (WC1/1) was previously shown to produce ciguatoxins and highly toxic maitotoxin-3 [8,22,23,24]. Ciguatoxins accumulate through marine food chains to cause the human poisoning known as ciguatera, whereas the role of maitotoxins in seafood poisoning is unclear [1,2]. In the early 1990s, we observed that the WC1/1 clone of *Gambierdiscus* produced a bimodal distribution of cell sizes in culture with cell pairs spontaneously fusing. It is now over 30 years since we first observed fusing cells with no other descriptions in the literature of such phenomena, only a brief pers. com. report of our results as “possible fusing gametes” [25]. Here, we provide a description of our observations to guide future research into the mechanisms of this phenomena in *Gambierdiscus* spp. and its implications for ciguatera.

## 2. Results and Discussion

### 2.1. A bimodal Cell Size Produced by Gambierdiscus in Culture

**Figure 1:**
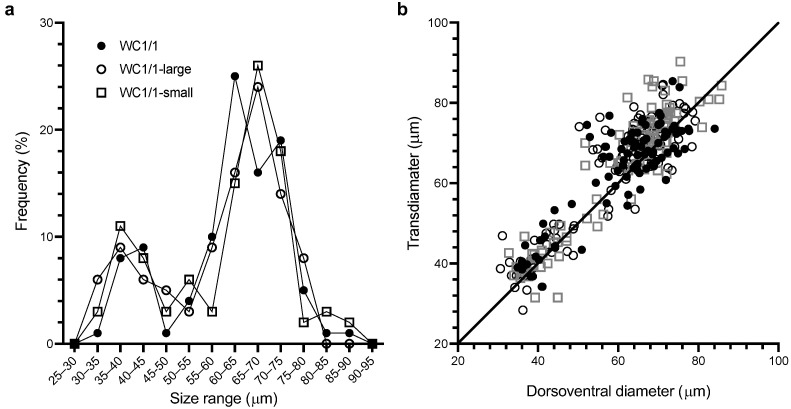
Cell size (µm) distributions for the dorsoventral and transdiameters of the WC1/1, WC1/1-large, and WC1/1-small clonal cultures of *Gambierdiscus*. (**a**) Frequency of dorsoventral diameters (*n* = 100 for each culture). (**b**) A plot of transdiameter vs. dorsoventral diameter for each of the cultures.

The WC1/1 clone of *Gambierdiscus* had a generation time of approximately 3 days (0.34 ± 0.03 divisions/day, *n* = 3) under the culture conditions used. These exponentially growing cultures contained mixtures of large and small sized cells that were consistent with a bimodal distribution of cell sizes (Figure 1). Once we realized cells were fusing in the WC1/1 culture, we searched for this phenomenon in all our other (~20) *Gambierdiscus* cultures. However, none of our other clonal cultures isolated from along the Queensland coast, or cultures isolated from French Polynesia, Hawaii, and the Virgin Islands [22] were observed to grow as a bimodal population under our culture conditions or show any evidence of fusing cells. To test the hypothesis that the culture might not have been clonal, we re-isolated clonal cultures from the original WC1/1 culture using the largest and smallest cells we could find, and then compared the sizes (dorsoventral diameters and transdiameters) of these new cultures (WC1/1, WC1/1-large, and WC1/1-small; Figure 1).

Non-parametric comparisons of the three WC1/1 cultures (Figure 1) revealed that there were no significant differences (*p* > 0.05) between the dorsoventral diameters or transdiameters of these cultures, respectively. This result is consistent with the original culture being clonal and that cell size was independent of the size of the inoculating cell. Similar concerns were dismissed by Bravo et al. [20] for their clonal culture of *Gambierdiscus* which produced a wide range of cell sizes. For all three of our cultures (WC1/1, WC1/1-large, and WC1/1-small), the dorsoventral diameters and the transdiameters were significantly different from normal distributions (*p* < 0.001 in all cases) but not significantly different from bimodal distributions (*p* > 0.05). The two modes for the measured cell diameters and the proportion of cells belonging to each mode were nearly identical for the re-isolated clonal cultures (Table 1).

We defined cells belonging to the smaller mode as <55 μm dorsoventral diameter (~28% of the pooled populations), while larger cells had dorsoventral diameters >55 μm (Table 1, Figure 1). There were indications that the lengths of WC1/1 cells were also bimodally distributed but the small sample sizes (a reflection of the difficulty of finding cells in girdle view) precluded statistical analysis. Bravo et al. [20] described finding gamete pairs from a clonal culture of *Gambierdiscus* producing a similar range of cell sizes (dorsoventral diameter range 32–77 µm) to that of our culture (Figure 1).

### 2.2. Cell Fusion (Light Microscope Observations)

**Figure 2:**
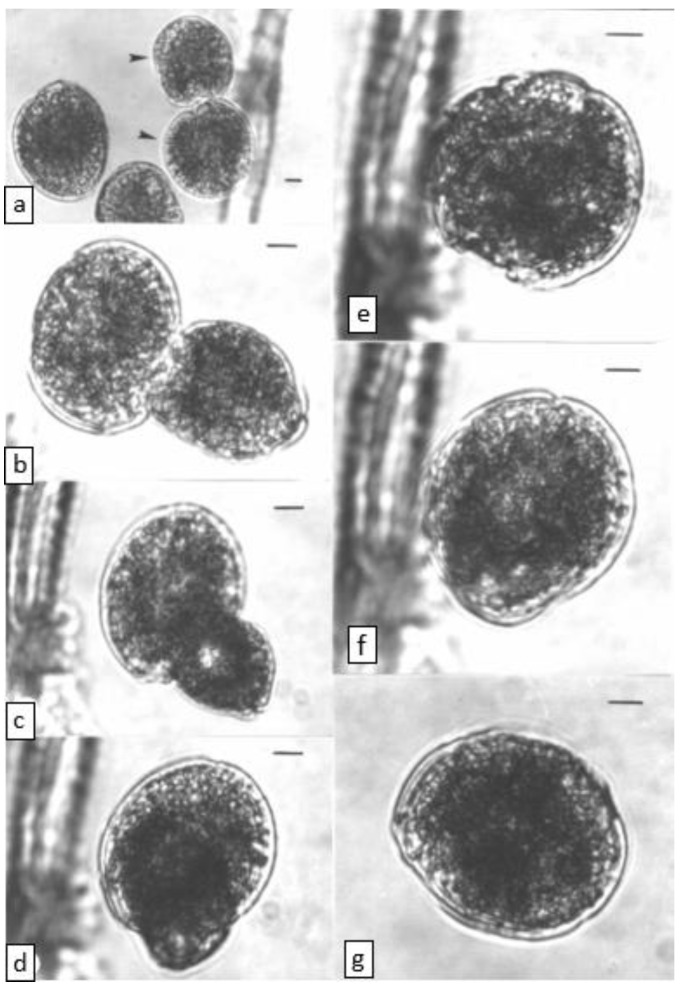
Time series for cell fusion in the WC1/1 clone of *Gambierdiscus*, scale bar = 10 µm. (**a**) time = 0 min. Fusing cells (arrows) orientating sulcus-sulcus, (**b**) time = 60 min. Fusing cells orientated at 90° relative to each other, (**c**) time = 75 min, (**d**), time = 99 min, (**e**) time = 108 min, (**f**) time = 116 min, (**g**) time = 144 min.

Cells from WC1/1 exponential and stationary phase cultures were frequently observed “dancing” and bumping into each other before fusing. Cell fusion was never observed in our other *Gambierdiscus* cultures (see Holmes et al. [22]). Fusion occurred throughout the light phase but appeared to be more prevalent a few hours after the onset of the light. Fusing cells were indistinguishable under the light microscope from other WC1/1 cells except that at least one (often both) of the fusing cells showed an extendible, finger-like protrusion (presumed peduncle) arising from near the sulcul region. This organ was considerably thicker and shorter than the longitudinal and transverse flagella and was capable of rapid movement. A peduncle was observed on many but not all WC1/1 cells and was not observed on cells from other cultures. This organ may have a sensory role as the initial orientation of fusing cells was always sulcus to sulcus (Figure 2) with the cells repeatedly bumping together about the sulcul regions, sometimes for more than an hour before fusing. During this time, no interconnections between the cells were seen and the peduncle did not appear to act as a cytoplasmic bridge between cells. One of the cells then turned 90° so that the planes of the girdles were approximately at right angles to each other, and the transverse flagella movement ceased in both cells, or in the cell seen in girdle (lateral) view. The two cells then began to fuse, with the cell in girdle view appearing to fuse into the theca of the other cell (Figure 2). The cell that had turned 90° often rounded up and became egg shaped (obovoid) during early fusion. Taylor [19] also suggested that two girdles can often be clearly seen in early fusion stages, sometimes at right angles to each other. Fusion appeared to initiate between the sulcul area of the cell turned on its edge and the area adjacent to the sulcus of the other cell (i.e., not directly sulcus to sulcus). Fusion can be quick (<10 min) or can take more than an hour, as occurred with the series shown in Figure 2. We saw no evidence of the theca being shed during fusion.

Fused cells have a lumpy, warty appearance but quickly round up and appear similar to other cells, except for a darker and denser appearance, especially towards the centre of the cell that may be diagnostic of the phenomena. This difference in colouration disappeared over a few days until the fused cell was indistinguishable from other cells by light microscopy. Similar sized cells (large and small) were observed fusing as well as dissimilar sized cells. Fusion of two small cells could produce a cell with larger area in apical view than the smaller cells from which it originated, or a cell not obviously larger in area. However, in the latter case, the cell may have had a longer length resulting in a more spherical cell shape. Occasionally small bubbles/droplets were released from the sulcul region during fusion. Fusion was not observed in any of our other *Gambierdiscus* clones, including cultures grown in high nutrient-f_2_ media that produced mainly “warty” shaped cells (see Holmes et al. [5]).

Cells spontaneously fused in WC1/1 cultures, but we have no evidence of nuclear fusion occurring, so we cannot be certain that these are observations of fusing gametes (sexual reproduction). The extendible proboscis which we have assumed is analogous to a peduncle [26] has not been reported previously in *Gambierdiscus* spp. It is possible that the peduncle has a role in cell fusion. Sensory roles have been suggested for peduncles that occur on both photosynthetic and non-photosynthetic dinoflagellate species [27] as well as facilitating feeding on prey (heterotrophy, myzocytosis) [28,29]. We did not observe the formation of a “copulation globule” [30] or cytoplasmic bridge/fertilization tube [31,32] between fusing *Gambierdiscus* cells.

An alternate hypothesis for fusing cells is that we could have been observing a form of mixotrophy (cannibalism). Mixotrophy is a common form of nutrition among photosynthetic dinoflagellates such as *Gambierdiscus*, and cannibalism has been reported for other dinoflagellate species [29,33]. Faust [34] was the first to suggest that *Gambierdiscus* can acquire nutrients through mixotrophy, and recent transcriptome analysis suggest that many dinoflagellates (including *Gambierdiscus*) retain an ancestral metabolic pathway for heterotrophic metabolism [35].

Cell fusion occurred spontaneously in the light cycle of both exponential and stationary WC1/1 growth phase cultures. Re-isolation of single small or large sized cells reproduced clonal cultures with the same bimodal population distribution of cell sizes as the initial clone. This indicates that it is unlikely that the original size distribution simply arose from isolation of a zygote (in which case the two re-isolated cells would also have had to be zygotes). We think it likely that the process of cell fusion contributed to the bimodal size of cultured populations, but we do not have evidence to confirm this. Nutrient deprivation is a common culture manipulation known to induce sexuality in autotrophic dinoflagellate species [18]. However, cell fusion occurred in both exponential and stationary growth phase WC1/1 cultures, making it unlikely that fusion was initiated in this strain by nutrient deprivation, especially since this clone was maintained for more than 8 years in f_10_ or f_10k_ media [22]. Cell fusion was never observed in our other cultures, many of which were grown for more than 10 years under the same conditions as the WC1/1 clone, including the same nutrient enriched seawater. This included the one other clone (NQ2/7) that we had in culture that produced ciguatoxins [22], making it unlikely that cell fusion is directly associated with the production of ciguatoxins.

### 2.3. Toxicity

**Figure 3:**
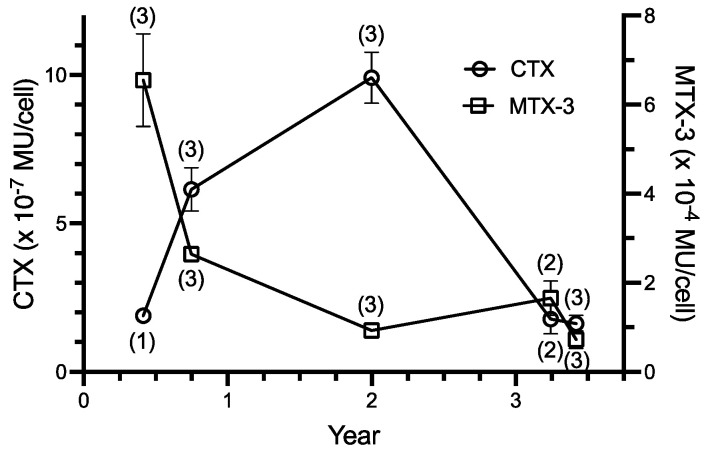
Production of pooled ciguatoxins (CTX) and maitotoxin-3 (MTX-3) by the WC1/1 clone of *Gambierdiscus* from the time the clone was isolated into culture in April 1988. Toxin concentrations expressed as means ± 1 standard error, MU = mouse unit, with *n* indicated in parentheses. Correlation for the 3 points in the first two years; r^2^ = 0.999.

The cellular yield of ciguatoxins extracted from the WC1/1 clone increased during the first two years in culture while there was a corresponding decrease in yields of maitotoxin-3 (Figure 3). During a further ~1.5 years in culture, ciguatoxin production decreased approximately five-fold from a maximum of 10^−6^ MU·cell^−1^ to a concentration similar to that detected in the initial extraction of this isolate. In contrast, maitotoxin-3 production declined approximately five-fold and then stabilized at between 1–2 × 10^−4^ MU·cell^−1^. Thus, there was no simple relationship between ciguatoxin and maitotoxin-3 production over the ~3.5 years of this study, although there was a strong inverse correlation for the first two years (Figure 3). Over this period, MTX-3 production declined exponentially while ciguatoxin levels showed a biphasic response, first increasing and later decreasing and never contributed >1% of total toxicity.

## 3. Materials and Methods

Cell sizes were measured from a clonal culture of the WC1/1 strain of *Gambierdiscus* isolated in April 1988 from Platypus Bay, adjacent to K’gari (Fraser Island) on the coast of Queensland, Australia [22]. Briefly, clonal cultures were started from single cells isolated using micropipettes which were maintained at 25 °C in f_10k_ medium under 50–60 μmol photon m^−2^ s^−1^ from Philips Daylight-54 fluorescent tubes with a 12:12 h light: dark photoperiod [5,22]. Cultures were maintained in 250 mL Erlenmeyer flasks and 50 mL Nunc plastic tissue flasks containing approximately 100 mL and 5 mL of f_10k_ media, respectively. Live cells (including fusing cells) were observed for extended periods of time in the tissue flasks using an inverted microscope (generally hours but sometimes fused cells remained in place and could be observed again next day). Cell dorsoventral diameters, transdiameters, and lengths (when possible) were measured from formalin (1–5%) preserved cells using an eyepiece micrometer and Olympus or Zeiss Jena light microscopes at a magnification of 600 times. Dorsoventral diameters and transdiameters were as measured by Holmes [36]. Dorsoventral diameters are sometimes termed cell depth, and transdiameters as cell width [20] or length [37]. We define length as the apical-antapical distance. Our discovery of fusing cells in the WC1/1 culture was facilitated by the routine culturing of cells in tissue culture flasks that made it easy for daily observation of undisturbed cells using an inverted microscope.

The WC1/1 clone produced a bimodal distribution of cell sizes containing large and small cells. The diameters of an exponentially growing culture of these cells were measured and then the largest and smallest cells were sorted under a dissecting microscope into two groups (named large and small, respectively) using a micropipette. Two new clones were started using the smallest and largest cells we could find, and the cell diameters of these new clones, termed WC1/1-large, and WC1/1-small, were then measured.

The WC1/1 clone was isolated at a time (1988) when only one species of *Gambierdiscus* was known (*G. toxicus*). Since then, at least 18 species of *Gambierdiscus* have been accepted in the literature [1] and therefore we cannot attribute the WC1/1 clone to a particular species. The clone was lost in the early 1990s after the fisheries program that hosted the research failed to secure ongoing support and after the authors had relocated to new research organizations. Previous attempts to publish these observations in the 1990s were rejected because we lacked the evidence to prove the process that was occurring with cell fusion (sexual reproduction, mixotrophy, or other). However, after more than 30 years, no comparable descriptions of fusing *Gambierdiscus* have been published.

Toxins were extracted, identified, and quantified from WC1/1 cultures in late exponential or early stationary growth phases after an average of 23 days growth (18–27 days), as previously described [8,22,23]. Toxins were quantified using mouse bioassay in mouse units (MU) because insufficient quantities of purified toxin were produced to weigh. One MU is the LD_50_ dose for a 20 g Quackenbush strain mouse. For ethical reasons, toxins were quantified using dose-death time curves to minimize the number of animals needed for acute toxicity testing [8,23], with all animal experiments carried out prior to 1994 under the then Australian National Health and Medical Research Council guidelines. Two ciguatoxins (named major and minor based upon toxin units) were produced by the WC1/1 clone [23] and their toxicity has been summed and expressed as total ciguatoxins. The major and minor ciguatoxins [23] were detected from all WC1/1 cultures except from the first extraction, when only the major ciguatoxin was detected. Maitotoxin-3 was detected from all WC1/1 cultures. The ciguatoxins reported here were identified based on their chromatography on silica gel, signs induced in mice, and pharmacological responses on isolated tissues [22,23]. Maitotoxin-3 was identified based on signs in mice and by high performance liquid chromatography and mass spectrometry [8,24].

Unless otherwise indicated, values are expressed as means ± 1 standard error with *n* = sample size. Cell population size frequencies were fitted to normal or bi-normal distributions and the proportion of the cell populations conforming to the bimodal means determined using the method of Macdonald and Pitcher [38]. Parametric (ANOVA) and non-parametric (Kolmogorov–Smirnov) comparisons of population sizes were carried out using Statistix (Analytical Software, MN). Data were also analysed using GraphPad Prism 9.3.1.

## Figures and Tables

**Table 1:** Comparison of dorsoventral and transdiameters from clonal WC1/1 *Gambierdiscus* cultures re-isolated from the original culture using the largest and smallest observed cells.

Clonal Culture	Dorsoventral Diameter (µm) and Percentage of Cells in Each Mode in Brackets		Transdiameter (µm) and Percentage of Cells Consistent with Each Mode in Brackets	
	Small Mode	Large Mode	Small mode	Large mode
WC1/1-large	40 ± 2 (28%)	66 ± 1 (72%)	42 ± 1 (29%)	70 ± 1 (71%)
WC1/1-small	39 ± 1 (26%)	67 ± 1 (74%)	41 ± 1 (28%)	71 ± 1 (72%)

## Data Availability

Data available on request.
